# *QuickStats*: Distribution* of Emergency Department Visits^†^ Made by Adults, by Age and Number of Chronic Conditions^§^ — United States, 2017–2019

**DOI:** 10.15585/mmwr.mm7101a6

**Published:** 2022-01-07

**Figure:**
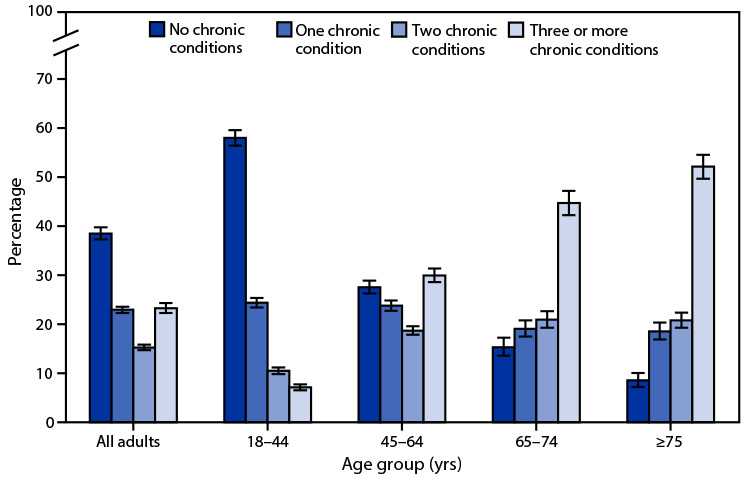
During 2017–2019, 38.5% of adult emergency department visits were made by patients with no chronic conditions, 22.9% made by those with one, 15.3% made by those with two, and 23.3% made by those with three or more chronic conditions. The percentage of adult emergency department visits made by patients with no chronic conditions or one chronic condition decreased with age, from 58.0% among patients aged 18–44 years to 8.5% among patients aged ≥75 years with no chronic conditions and from 24.4% among patients aged 18–44 years to 18.5% among patients aged ≥75 years with one chronic condition. In contrast, the percentage of visits by patients with two or three or more chronic conditions increased with age, from 10.5% among patients aged 18–44 years to 20.8% among patients aged ≥75 years with two conditions and from 7.1% among patients aged 18–44 years to 52.1% among patients aged ≥75 years with three or more chronic conditions.

**Source**: The National Center for Health Statistics, National Hospital Ambulatory Medical Care Survey, 2017–2019. https://www.cdc.gov/nchs/ahcd/ahcd_questionnaires.htm

